# A Czech version of the Overall Anxiety Severity and Impairment Scale (OASIS): standardization and psychometric properties

**DOI:** 10.1186/s12888-022-04365-5

**Published:** 2022-12-23

**Authors:** Petr Mikoska, Lukas Novak, Lubor Pilarik, Tomas Bok, Martin Fulep, Richard Korinek

**Affiliations:** 1 Olomouc University Social Health Institute, Palacky University in Olomouc, Univerzitni 244/22, 771 11, Olomouc, Czech Republic; 2 Department of Community and Occupational Medicine, University Medical Center Groningen, University of Groningen, Groningen, the Netherlands; 3 Faculty of Education, Matej Bel University in Banská Bystrica, Banská Bystrica, Slovak Republic

**Keywords:** Anxiety, OASIS, Assessment, Psychometrics, Measurement

## Abstract

**Background:**

The Overall Anxiety Severity and Impairment Scale (OASIS) is a transdiagnostic measure that assesses severity and impairment associated with anxiety disorders. However, its psychometric properties were primarily examined in English-speaking or western countries. Therefore, this study aims to examine its psychometric parameters in the Czech Republic.

**Methods:**

A large representative sample (*n* = 1738), a clinical sample (*n* = 57) and a retest sample (*n* = 20) were used. In addition to the OASIS, conventional measures of anxiety, depression, personality traits, self-esteem, life satisfaction, and other scales were also administered. Moreover, we examined the latent structure, reliability, validity, and the cut-off score for the Reliable Change Index (RCI) and the Clinically Significant Change Index (CSI).

**Results:**

Higher anxiety was found in females, religious non-church members, and students. The Confirmatory Factor Analysis supported the adequate fit of the unidimensional solution: x2(4) = 3.20; *p* < 0.525; CFI = 1.000; TLI = 1.000;RMSEA = 0, SRMR = 0. The measurement equivalence examination indicated that the OASIS measures anxiety invariantly between males and females. The validity of the OASIS was supported by positive associations with neuroticism, depression, perceived stress, guilt, shame, and the established anxiety measures. The internal consistency was excellent (Cronbach’s alpha = 0.96, McDonald’s omega = 0.96). The test-retest reliability was acceptable (*r* = 0.66). The cut-off for the CSI is 6 and the RCI is 5.32.

**Conclusions:**

The OASIS represents a reliable and valid instrument for assessing anxiety in adults. Due to its shortness, excellent psychometric properties, and percentile norms, it is especially useful for short and accurate screening of anxiety and mapping therapeutic changes in clinical practice.

**Supplementary Information:**

The online version contains supplementary material available at 10.1186/s12888-022-04365-5.

## Introduction

Anxiety disorders are considered the most common psychiatric diagnosis [1]. The development of an anxiety disorder is a risk factor for the emergence of other anxiety or mood disorders and addictions. The presence of comorbidity complicates the treatment of coexisting disorders, leads to a worse prognosis, and increases the risk of suicide. Furthermore, an untreated anxiety disorder is associated with impaired functioning in the social area (i.e., impaired interpersonal relationships, decreased work performance, unemployment, and phobic avoidance [2]). Moreover, their prevalence tends to grow. For instance, compared to 2017, in May 2020 (in the first wave of the COVID-19 pandemic), there was an increase in the prevalence of anxiety disorders by 7% [3, 4]. Moreover, in November 2020 (i.e., the second wave of the COVID-19 pandemic), the prevalence of anxiety disorders almost doubled compared to November 2017 [3, 5]. Such an increase in the prevalence of anxiety disorders suggests that their treatment might not only improve population health [6, 7] but can also significantly increase economic productivity [8]. In order to facilitate diagnostics and treatment of anxiety disorders, there is a great need for short and transdiagnostic measuring tools, which would differentiate patients who need a specialized intervention while allowing psychotherapists to monitor the effectiveness of interventions over relatively short periods of time [9, 10].

Current studies [9, 11] show the importance of monitoring the functionality of emotions (i.e., to what extent emotions affect clients’ life) more than merely observing the degree of emotional experience. To determine the degree of anxiety, the Generalized Anxiety Disorder Questionnaire [12], the Beck Anxiety Inventory [13], and the State-Trait Anxiety Inventory [14] have been used nationwide. Although all these tools capture the general severity of anxiety, they suffer from several weaknesses, making their use limited. First, they do not focus so much on changes in an individual’s functioning e.g. in the work or social environment [15]. Second, these traditional self-assessment scales are usually based on the assumption that the frequency of symptoms also reflects their severity, thus ignoring the aforementioned key aspect of a disorder [11]. Third, some of the above mentioned measures are relatively lengthy, and thus create a burden, not only for administrators themselves but especially for clients [9, 15].

The Overall Anxiety Severity and Impairment Scale (OASIS), developed by Norman, et al., [16], responds to the limits associated with existing screening tools. This five-item questionnaire captures the severity of all types of anxiety disorders and, due to its brevity, minimizes the burden on the respondents [17]. A great advantage is that the scale also considers the functional impairments of the respondent associated with the level of experienced anxiety [15]. Moreover, previous validation studies of the OASIS repeatedly supported the factorial validity and showed excellent psychometric properties, as evidenced by, for example, the value of the Cronbach’s alpha coefficient, which ranges from 0.80 to 0.94 and indicates good internal consistency [9, 15, 16, 18, 19].

However, the psychometric qualities of the OASIS were identified mostly in English-speaking or western countries. Therefore, this study aims to examine its psychometric parameters in the Czech Republic. We want to present the first instrument assessing functional impairment associated with anxiety in Czech culture. Our goal is to find out whether the OASIS assesses anxiety similarly compared to abroad, and if it represents an applicable transdiagnostic instrument for screening the psychotherapeutic process in our socio-cultural context. The specific objectives were (1) testing of the basic psychometric properties (i.e., factor structure, reliability, including test-retest reliability and validity) of the OASIS in the Czech population; (2) verification of measurement invariance between males and females; (3) determination of the cut-off value for the Reliable Change Index (RCI) and the Clinically Significant Change Index (CSI); (4) creation of Czech percentile norms for the OASIS based on representative data. Contingent on the above objectives of this study, we tested the hypotheses presented in the pre-registration form (https://osf.io/yxm9g).

## Methods

To explore the psychometric parameters of the OASIS, we collected data from 3 different samples. In each sample, we performed a quality check and an outlier screening. This screening aimed to explore whether extreme responses, detected by the Median Absolute Deviation (MAD), did not contain a uniform pattern of responding (i.e., the same answers to multiple different questions). If the uniform pattern of responding was observed, the participant was removed from a dataset. For more details about how the MAD was calculated see the pre-registration form (https://osf.io/yxm9g).

Participants were informed about the study goals in all surveys, and they provided informed consent prior to the data collection. All subjects were told that their participation was anonymous and entirely voluntary. They were also instructed that they could leave the survey at any moment. The study was approved by the Ethics Committee of the Olomouc University Social Health Institute, Palacký University Olomouc (No. 2021/6), and all methods were performed in accordance with the relevant guidelines and regulations.

For the purposes of this study, the following inclusion criteria were stated: Czech adults ≥ 18 years of age for the non-clinical population and Czech adults ≥ 18 years of age in outpatient/inpatient psychiatric or psychological care for the clinical population. Participants were excluded if they provided inaccurate responses, refused to give informed consent, or were unwilling to participate in the study. Participants who were unwilling to fill out the retest questionnaires were also removed from the sample.

There are several reasons why we recruited three different samples: first, we needed a representative sample consisting primarily of healthy subjects to construct population norms. Second, inpatient and outpatient samples were necessary to define cut-off scores for Reliable Change Index and Clinically Significant Change Index. Third sample was needed for the purpose of establishing test-retest reliability.

### Participants

#### Sample 1

This sample consisted of Czech participants from the national representative sample above 18 years of age. The stratified random sampling method was used to segment respondents based on sex, age, and region of origin. In total, 2032 participants were asked to participate in the survey. Of these 2032 subjects, 1738 (87.1%) participated in the survey. The most frequent reason for rejection of participation in the survey was a lack of time (38.9%), followed by an unwillingness to participate (26.3%). The data collection took place between September and November 2020. The OASIS was administered by specially trained investigators (*n* = 216) via the face-to-face Standardized Survey Interviewing method. No missing values were present. The outlier screening suggested that no participant answered uniformly. Participants with age lower than 18 years were excluded (*n* = 31). Thus, the final number of subjects was 1738 (Age: *M* = 47.75, *SD* = 17.60, Females: 51.38%).

#### Sample 2

The clinical sample was collected between July and October 2021. The survey was conducted on outpatients and inpatients above 18 years of age. Patients had various mental disorders (for the number and percentages of individual diagnoses, see the online Supplementary Table 1). Participants had to fill out a questionnaire administered by a psychologist via the paper-and-pencil method. All participants were Czech adults. Of the initial 61 subjects, we removed participants (*n* = 2) with low quality responses, resulting in 59. We further removed participants with age lower than 18 years (2) which resulted in 57 (Age: *M* = 35.56, *SD* = 14.95, Females: 70%).

#### Sample 3

Participants consisted of Czech adults recruited by the convenience sampling method for purposes of the test-retest reliability. The online data collection took place between November and September 2021. From the initial 41 participants, we removed those who did not fill out the questionnaire a second time, resulting in 21 subjects. We also removed participants (*n* = 1) with low quality responses, resulting in 20 participants (Age: M = 39.80, SD = 11.10, Females: 65%). A detailed description of the study samples can be found in Table 1.

**Table 1:** Socio-demographic characteristics of the study samples

Value	Sample 1	OASIS: M(SD) - Sample 1	Sample 2	OASIS: M(SD) - Sample 2	Sample 3	OASIS: M(SD) - Sample 3
Gender						
Male	845 (49%)	1.95 (3.05)	16 (28%)	6.56 (3.42)	7 (35%)	2 (2.31)
Female	893 (51%)	2.96 (3.44)	41 (72%)	10.12 (4.52)	13 (65%)	2.62 (2.99)
Family_status						
In partnership/married	875 (50%)	2.26 (3.03)	28 (50%)	9.57 (4.48)	16 (80%)	2.81 (2.88)
Not married	460 (26%)	2.6 (3.49)	21 (38%)	9.33 (4.68)	2 (10%)	1.5 (0.71)
Divorced	222 (13%)	2.32 (3.15)	7 (12%)	5.67 (3.27)	2 (10%)	0 (0)
Widow/widower	181 (10%)	3.32 (3.98)				
Education						
Basic school	118 (6.8%)	3.15 (4.18)	2 (3.5%)	9.5 (2.12)		
Vocational school or without graduation high school	512 (29%)	2.3 (3.18)	9 (16%)	9.89 (6.19)		
High school or higher vocational school	714 (41%)	2.56 (3.39)	23 (40%)	9 (4.5)		
University	394 (23%)	2.31 (2.92)	23 (40%)	8.86 (4.11)		
Economical_status						
Mental worker (medical doctor..)	311 (18%)	2.01 (3.02)				
Entrepreneur	108 (6.2%)	1.82 (2.72)	4 (7.7%)	6.75 (2.5)	7 (35%)	1.71 (1.5)
Manual worker	234 (13%)	1.63 (2.76)				
Unemployed	73 (4.2%)	2.81 (3.59)	4 (7.7%)	8.25 (6.34)	2 (10%)	4 (0)
Employee unspecified	59 (3.4%)	1.19 (2.26)	23 (44%)	9.45 (4.92)	10 (50%)	2.7 (3.59)
Administrative worker	161 (9.3%)	2.68 (3.19)				
Employee in services	300 (17%)	2.88 (3.35)				
Student	126 (7.2%)	3.13 (3.77)	11 (21%)	8 (3.44)	1 (5.0%)	1 (NA)
Pensioner	366 (21%)	3.07 (3.65)	10 (19%)	10.7 (4.08)		
Religiosity						
Religious, member of a church	187 (11%)	2.56 (3.38)	6 (11%)	10.17 (6.49)		
Religious, not member of a church	414 (24%)	2.89 (3.48)	19 (34%)	8.67 (4.12)		
Not religious	1,137 (65%)	2.3 (3.2)	31 (55%)	9.23 (4.49)		
Income_of_family						
< 10 000	20 (1.2%)	1.9 (2.59)				
10.001-20.000	259 (15%)	3.09 (3.77)				
20.001-30.000	380 (22%)	2.58 (3.3)				
30.001-40.000	372 (21%)	2.35 (3.12)				
40.001-50.000	281 (16%)	2.18 (3.1)				
50.001-60.000	213 (12%)	2.38 (3.14)				
60.001-70.000	110 (6.3%)	2.26 (3.25)				
70.001+	103 (5.9%)	2.27 (3.47)				
Age mean and standard deviation	47.75 (17.6)		35.56 (14.95)		39.8 (11)	
Number of participants	1738		57		20	

*SD* standard deviation, *M* mean, *OASIS* Overall Anxiety Severity and Impairment Scale, Income of family is expressed in Czech crowns

### Measures

There are a large number of scales capturing various specific symptoms of anxiety disorders. Since the manifestations of anxiety can be greater in comorbidity, we decided to include a larger number of questionnaire methods capturing anxiety symptoms in our research [15]. During the compilation of the battery of tests, we also included instruments assessing constructs (e.g., neuroticism, well-being, life satisfaction) that usually overlap with anxiety disorders [15].

The Overall Anxiety Severity and Impairment Scale (OASIS) [16]. The scale contains five items. These items are related to the frequency of anxiety symptoms and their intensity as well as interference with the person’s work or school life and social life. All items are rated on a 5-point Likert scale, ranging from 0 to 4. Higher scores indicate greater severity and functional impairment as a result of anxiety symptoms [16]. For this study, we used the Czech translation of the original scales (Appendix 1). The translation process followed the translation guidelines of the WHO [20] (forward and backward translation, expert panel, qualitative interview). To ensure a proper understanding of the term “anxiety” for all participants, the definition of this construct was administered together with the questionnaire items.

The Patient Health Questionnaire (PHQ-9) [21], validated in the Czech Republic by Daňsová et al., [22], is a widely used self-report measure for depressive symptomatology screening. The PHQ-9 is a nine-item method in which participants answer questionnaire items on a 4-point Likert scale, ranging from 0 (“Not at all”) to 3 (“Nearly every day”). The items are based directly on the nine signs and symptoms of a depressive disorder, according to the DSM-IV criteria [21]. A higher score indicates a higher degree of depression. A more contemporary study [23] shows very good test-retest reliability (*r* = 0.86) of the PHQ. The internal consistency of the PHQ-9 in the clinical sample is good with Cronbach’s alpha $\alpha$ = 0.92, 95% CI [0.89–0.95] and McDonald’s ${\omega }_{t}$ = 0.92, 95% CI [0.89–0.95]. The construct validity is supported by a significant positive correlation with the PSS-10 (*r* = 0.67) and a negative correlation with the SCS (*r* = -0.64).

The Generalized Anxiety Disorder Questionnaire (GAD-7) [12]. Psychometric characteristics of the Czech version of the GAD-7 were tested by Prikner [24]. The GAD-7 is a 7-item self-report measure in which items are rated on a 4-point Likert-type scale (0 = “not at all” to 3 = “nearly every day”). The GAD-7 items describe some of GAD’s most salient diagnostic features (i.e., feeling nervous, anxious, or on edge and worrying too much about different things). Higher scores indicate more severe GAD symptoms. In one of the more recent studies [25], the GAD-7 demonstrates reasonable temporal stability (*r* = 0.87). Cronbach’s alpha and McDonald’s omega for the GAD-7 in the clinical sample are $\alpha$ = 0.91, 95% CI [0.88–0.95] and ${\omega }_{t}$ = 0.91, 95% CI [0.87–0.95], respectively. There is a significant positive correlation between the GAD-7 and the PSS-10 (*r* = 0.62) which indicates adequate convergent validity.

The Rosenberg´s Self-Esteem Scale (RSES) [26]. Is a 10-item self-report measure, assessing a person’s overall worthiness as a human being [26]. A higher score is indicative of higher global self-esteem. The Czech version of the RSES was validated by Blatný et al., [27]. Responses are coded on a 4-point Likert-type scale, ranging from 1 (“strongly disagree”) to 4 (“strongly agree”). Correlations of 0.85 and 0.88 display excellent stability of the RSES in a more recent research [28]. Cronbach’s alpha and McDonald’s omega for the RSES in the clinical sample are $\alpha$ = 0.87, 95% CI [0.81–0.92] and ${\omega }_{t}$ = 0.87, 95% CI [0.81–0.92], respectively. The strong negative association with the GSES (*r* = -0.81) and the strong positive correlation with the SCS (*r* = 0.68) support reasonable construct validity of the RSES.

The Big Five Inventory (BFI) [29]. The czech version was validated by Hrebickova et al., [30]. The BFI is a 44-item self-report instrument used to measure the five personality domains according to the Five-factor model. We used subscales Neuroticism (N), Openness (O), and Agreeableness (A), which include self-descriptive statements that participants respond to using a 1 (“strongly disagree”) to 5 (“strongly agree”) Likert-type scale. Higher scores indicate higher neuroticism, agreeableness, and openness. Recent psychometric research of the BFI shows reasonable test-retest correlations (> 0.75) in two subsamples [31]. Cronbach’s alpha and McDonald’s omega for the N in the clinical sample are $\alpha$ = 0.87, 95% CI [0.82–0.92] and McDonald’s ${\omega }_{t}$ = 0.88, 95% CI [0.83–0.93], for O $\alpha$ = 0.87, 95% CI [0.83–0.92], McDonald’s ${\omega }_{t}$ = 0.88, 95% CI [0.84–0.93] and for A $\alpha$ = 0.48, 95% CI [0.29–0.68], McDonald’s ${\omega }_{t}$ = 0.55, 95% CI [0.39–0.71]. The BFI (N) positively correlates with the PSS-10 or the GSES. In contrast, the BFI (A) negatively correlates with the PSS-10. The BFI (O) negatively correlated with PHQ-9 and GAD-7. These correlations may support construct validity of the BFI subscales.

The Perceived Stress Scale-10 (PSS-10) [32]. The PSS-10 is a self-report measure designed to assess the degree of stress induced by daily life situations. The Czech version of the PSS-10 was validated by Buršíková and Kohout [33]. Participants answer the scale items on a 5-point Likert-scale, ranging from 0 (“never”) to 4 (“very often”). A higher total score indicates a higher level of perceived stress. A shorter version of the PSS (i.e., PSS-10) demonstrates adequate test-retest reliability (*r* = 0.7) [34]. Cronbach’s alpha and McDonald’s omega for the PSS-10 in the clinical sample are $\alpha$ = 0.88, 95% CI [0.83–0.92] and ${\omega }_{t}$ = 0.88, 95% CI [0.83–0.93], respectively. The significant positive correlations between the PSS-10 and the GAD-7 or the PHQ-9 show high convergent validity of this questionnaire.

The Guilt and Shame Experience Scale (GSES) [35] is a self-report measure of one’s experiences of guilt and shame. The GSES items are divided into a guilt subscale and a shame subscale, each containing four items. For each item, respondents answer questions using a four-point scale, ranging from 1 (“not at all”) to 4 (“significantly”). A higher score corresponds to higher experiences of guilt and shame. Cronbach’s alpha and McDonald’s omega for the GSES in the clinical sample are $\alpha$ = 0.90, 95% CI [0.86–0.94] and ${\omega }_{t}$ = 0.90, 95% CI [0.86–0.94], respectively. The significant negative correlation (*r* = -0.75) between the GSES and the SCS may support construct validity of the method.

The Self-Compassion Scale (SCS) [36] is a 26-item instrument. The Czech version of the SCS was validated by Benda and Reichova [37]. Participants rate each item on a 5-point Likert scale from 1 (“almost never”) to 5 (“almost always”). A higher score is indicative of higher self-compassion. In recent research [38], the longer (26-item) version of the SCS showed a test-retest reliability coefficient of 0.92. This value indicates excellent stability Cronbach’s alpha and McDonald’s omega for the SCS in the clinical sample are $\alpha$ = 0.92, 95% CI [0.89–0.96] and ${\omega }_{t}$ = 0.92, 95% CI [0.89–0.95], respectively. In the present study, we have found a significant positive relationship between the SCS and the RSES. In contrast, there is a weak association with the Openness (BFI). These correlations can support construct validity of the scale.

The Patient Health Questionnaire Somatic Symptom Severity Scale (PHQ-15) [39] is a self-administered questionnaire consisting of 15 items oriented to severity of somatic symptoms. The scale is usually used to capture symptoms of somatoform disorders defined according to the DSM-IV [40]. The symptoms should be present for at least 4 weeks and their severity is rated on a 3-point Likert scale from 0 (“not bothered at all”) to 2 (“bothered a lot”) [39]. In one of the more contemporary studies [41], the test–retest reliability was acceptable (i.e., *r* = 0.65).Cronbach’s alpha and McDonald’s omega for the PHQ-15 in the present sample are $\alpha$ = 0.82, 95% CI [0.74–0.90] and ${\omega }_{t}$ = 0.82, 95% CI [0.75–0.90], respectively. In this study, the PHQ-15 also positively correlates with the PSS-10 and the SWLS, but there is a small association with the SCS. These associations provide evidence of the construct validity of this tool.

The Clinical Outcomes in Routine Evaluation (CORE OM) [42] is a self-report inventory consisting of 34 items focused on the frequency of difficulties in the last seven days. The CORE OM consists of four subscales [42]. However, for this study, we used only three of them: (1) subjective well-being (CORE OM W), (2) problems (CORE OM P), (3) functioning (CORE OM F). The CORE OM and its subscales show great psychometric properties and sensitivity to change in psychotherapy, as was originally intended [42]. The psychometric properties of the Czech version of the CORE OM were tested by Seryjová et al., [43]. A newer Swedish validation study [44] demonstrated that temporal stability of the CORE-OM subscales was good. The intra-class correlations were 0.80 for subjective well-being, 0.80 for problems and 0.77 for functioning. All subscales yielded excellent internal consistency: well-being: $\alpha$ = 0.79, 95% CI [0.69–0.88], ${\omega }_{t}$ = 0.82, 95% CI [0.74–0.89], functioning subscale: $\alpha$ = 0.86, 95% CI [0.82–0.91], ${\omega }_{t}$ = 0.90, 95% CI [0.86–0.94] as well as problems subscale: $\alpha$ = 0.90, ${\omega }_{t}$ = 0.93. We have found a significant positive association between the CORE OM (W, F) and the SWLS. There are also negative correlations between the CORE OM (P) and the PHQ-9 or the GAD-7. These associations support convergent validity of the CORE OM.

The Satisfaction With Life Scale (SWLS) [45] is a brief 5-item scale designed to measure the subjective level of life satisfaction [46]. The statements are rated on a 7-point scale indicating the degree of agreement (from 1 = strongly disagree to 7 = strongly agree) with each statement [47]. The overall level of life satisfaction is expressed as a sum of points for all items [37]. The adequate psychometric properties of the Czech version were supported by Lewis et al., [48]. A high correlation (*r* = 0.86) between two administrations of the SWLS was found and indicates very good test-retest reliability of this scale [49]. Cronbach’s alpha and McDonald’s omega for the SWLS in the present sample are 0.88 and 0.88, respectively. The convergent validity of the SWLS can be supported by a positive association with the RSES and the CORE OM subscales (*r* = 0.59, 0.65, 0.63). The good discriminating ability of the SWLS shows a weak positive correlation (*r* = 0.12) with the Openess (BFI).

### Data analysis

Testing of statistical assumptions was performed on each sample. If no sample is specified, the results of this testing apply to all three samples. Multivariate kurtosis and skewness of the OASIS items were inspected by the Mardia test, which suggested that the normality assumption was violated. Therefore, statistical tests that do not assume the Gaussian distribution were used. Visual screening of the residual plot suggested slight heteroscedasticity (Sample 1), which was, however, not supported by the Breusch-Pagan test (${\chi }^{2}$ = 5.08, *df* = 1, *p* = 0.024).

To explore the dimensionality of the OASIS, the Confirmatory Factor Analysis (CFA) on the first sample (*n* = 1738) was performed. Only a one-factor model was evaluated as the OASIS was developed as an unidimensional scale. The power analysis suggested that at least 1021 participants were needed for the CFA. More details about the power analysis procedure can be found in the pre-registration form (https://osf.io/yxm9g). The adequacy of the correlation matrix was assessed by the Bartlett test and the Kaiser Meyer Olkin (KMO) measure. The absolute model fit was explored by the following indicators: the Standardized Root Mean Square Residual (SRMR) and the Root Mean Square Error of Approximation (RMSEA). In these indicators, values < 0.08 suggest an acceptable fit and values < 0.05 a good fit [50–53]. The Comparative Fit Index (CFI) and the Tucker-Lewis Index (TLI) were used to evaluate the incremental fit of the model. Values of the TLI and the CFI > 0.95 suggest an acceptable fit [54] and values > 0.97 a good fit [55]. The covariance implied by the model was compared with the covariance observed by the chi-square test. With manifest variables having five categories, based on the recommendation of [56], we used the Diagonally Weighted Least Squares estimator (DWLS) to fit our model. Polychoric correlations were also utilized during the fitting procedure.

Measurement equivalence was tested via the multigroup CFA. The invariance of the measurement was explored in terms of gender (males, females) in the first sample. If the $\varDelta$ CFA was > 0.01, then a good fit of a nested model was rejected. The internal consistency of the OASIS was assessed by Cronbach’s alpha and McDonald’s omega. Convergent, divergent, and concurrent validity were inspected by the zero-order Spearman correlation coefficient (Sample 2) and analysis of covariance (ANCOVA). In the ANCOVA (Sample 2), the outcome variable was gender, the predictor was the OASIS score, and neuroticism was the covariate. The temporal stability of the OASIS score was examined by a two-way random effects intraclass correlation coefficient. The time interval between the first and the second administration of the OASIS was seven days (Sample 3). To identify the cut-off value for the clinically significant change (CSC), we used the [57] formula, where SD_0_ and M_0_ are the standard deviation and the mean of the non-clinical sample, while SD_1_ and M_1_ are the standard deviation and the mean of the clinical sample. This formula can be mathematically expressed as follows:

$$c=\frac{S{D}_{0}{M}_{1}+S{D}_{1}{M}_{0}}{S{D}_{0}+S{D}_{1}}$$

We also examined an optimal cut-off for statistically significant change - the Reliable Change Index (RCI). This cut-off was determined based on a modified version of the [57] formula. The modified version presented by [58] and can be expressed as follows:

$$RCI=1.96\sqrt{2 S{D}^{2}\left(1-rel\right)}$$

Differences between socio-demographic groups in the OASIS (Sample 1) were examined via the Wilcoxon signed rank test and the Kruskal–Wallis test (non-parametric ANOVA). The post-hoc analysis consisted of the Dunn test and the Games-Howell test. The degree of an effect for socio-demographic comparison was estimated by the Vargha and Delaney $\widehat{A}$ [59], in which values of $\widehat{A}$ between 0.56 and 0.64 indicate a small effect, 0.64–0.71 a medium effect, and > 0.71 a large effect. In order to create population norms, we used polynomial regression to capture three-dimensional relationships between the norm score, explanatory variable (i.e., age), and expected raw score of the OASIS [60]. Descriptive and inferential statistics procedures were done in R [Version 4.2.1; [61]] using the following libraries: *lavaan* [62], *papaja* [63] *psych* [64], *usf* [65], *cNORM* [60].

## Results

### Socio-demographic results

The results revealed that females reached a significantly higher score in the OASIS compared to males (W = 303,623.50, *p* < 0.001, *r* = 0.18). In addition, religious non-church members reached a significantly higher score in the OASIS compared with non-religious individuals (t(683.80) = 3, *p* = 0.008, $\widehat{A}$ = 0.55). Furthermore, there were differences in anxiety in the family status: widows and widowers had significantly higher levels of anxiety compared to those who were married or in a partnership (t(339.33) = 2.75, *p* = 0.032, $\widehat{A}$ = 0.43). In the socio-economic status, we found significantly higher anxiety levels in administrative workers (t(310.86) = 3.38, *p* = 0.023, $\widehat{A}$ = 0.40), employees in services (t(530.39) = 4.73, *p* < 0.001, $\widehat{A}$ = 0.$\widehat{}$37), pensioners (t(581.68) = 5.47, *p* < 0.001, $\widehat{A}$ = 0.38), and students (t(198.77) = 3.92, *p* = 0.004, $\widehat{A}$ = 0.38) compared to manual workers. Similarly, pensioners had a significantly higher score in the OASIS compared to mental workers (t(674.50) = 4.12, *p* = 0.001, $\widehat{A}$ = 0.41).

### Confirmatory factor analysis results

The results of the Bartlett test (${\chi }^{2}$ (10) = 6,432.31, *p* < 0.001) and the KMO indicated that our data met the assumptions of the factor analysis. The CFA (Sample 1) suggested that although the relative fit indices indicated a good fit of the unidimensional solution, some absolute model fit indices, i.e., the RMSEA yielded inadequate values: ${\chi }^{2}$(5) = 108.62, *p* < 0.001, CFI = 1.00,TLI = 1.00,SRMR = 0.03, RMSEA = 0.11, 90% CI [0.09–0.13]. Based on an inspection of the modification indices, constraints between items 1 and 2 were released. This aligns with the approach and theoretical reasons outlined in other psychometric studies of the OASIS [9, 11, 15].

**Fig. 1:**
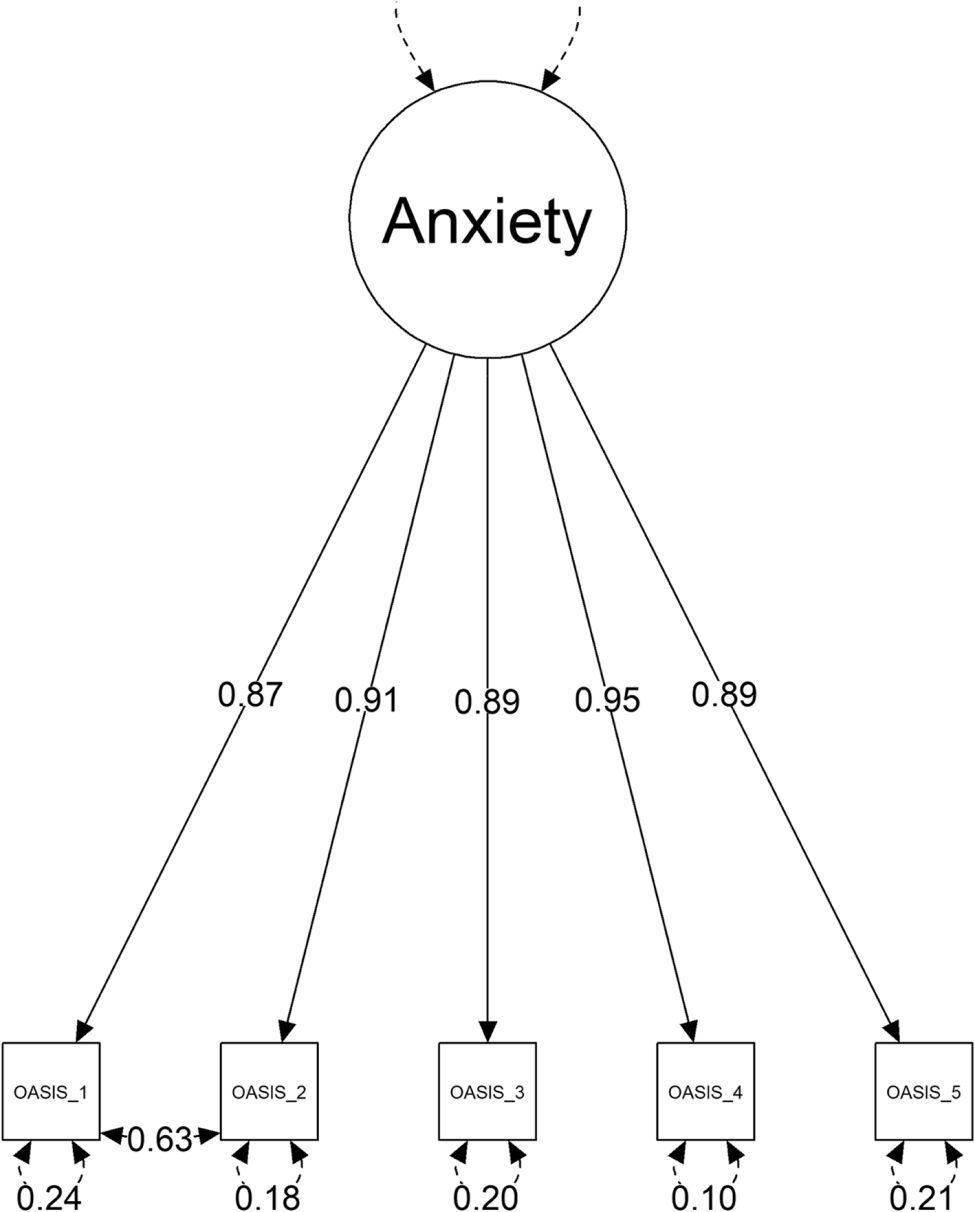
depicts a Structural Equation Model (SEM) diagram. The term “anxiety” inside a circle represents a latent variable. The OASIS items inside a rectangular box represent manifest variables. Numbers inside a solid line arrows represent factor loadings. Double arrow between OASIS_1 and OASIS_2 represents correlation between errors of these two variables. Double arrows below individual manifest variables represent measurement errors (*n* = 1738)

After a modification of the base model, the fit indices yielded excellent values: ${\chi }^{2}$(4) = 2.97, *p* = 0.563, CFI = 1,TLI = 1,SRMR = 0.00, RMSEA = 0, 90% CI [0-0.03]. The chi-square difference test supported a better fit of the model with the correlated residuals: ${\chi }^{2}$(1) = 107; *p* < 0.001. In the modified model, the correlation between the item residuals was low with a maximum value of 0.01. The factor loadings of the manifest variables were high (Fig. 1).

### Invariance testing and factor loadings

The change in the CFI < 0.01 across configure, metric, scalar, and strict models supports the measurement invariance of the OASIS between the two genders (Table 2).

**Table 2:** Measurement equivalence of the OASIS between genders (*n* = 1738)

Model	x2	df	p-value	CFI	TLI	RMSEA (90% CI)	SRMR
Overall model	0.039	1	0.843	1	1	0 (0-0.037)	0.001
Male model	0.161	1	0.688	1	1	0 (0-0.068)	0.002
Female model	0.034	1	0.853	1	1	0 (0-0.049)	0.001
Configural model	0.195	2	0.907	1	1	0 (0-0.027)	0.002
Metric model	2.473	5	0.781	1	1	0 (0-0.031)	0.006
Scalar model	9.184	16	0.906	1	1	0 (0-0.013)	0.002
Strict model	9.184	16	0.906	1	1	0 (0-0.013)	0.002

*x2*: chi-square, *df*: degrees of freedom, *CFI*: Comparative Fit Index, *TLI*: Tucker-Lewis index, *RMSEA*: Root Mean Square Error of Approximation, *CI*: Confidence Interval, *SRMR*: Standardized Root Mean Square Residual

### Internal consistency and item statistic

The OASIS yielded an excellent internal consistency in the representative sample: with Cronbach’s $\alpha$ = 0.96, 95% CI [0.96–0.96] and McDonald’s ${\omega }_{t}$ = 0.96, 95% CI [0.96–0.96], as well as in the clinical sample: Cronbach’s $\alpha$ = 0.92, 95% CI [0.88–0.95] and McDonald’s ${\omega }_{t}$ = 0.92, 95% CI [0.88–0.95]. Table 3 depicts statistics for the OASIS items. Overall, the correlations between these items and item-total correlations were high. OASIS 5 had the lowest correlation with other scale items. This item also had the lowest item-total correlation.

**Table 3:** Polychoric correlations of the OASIS items with SD, M and ITC (*n* = 1738)

OASIS_1	OASIS_2	OASIS_3	OASIS_4	OASIS_5	M	SD	ITC	Skewness	Kurtosis
1					0.69	0.87	0.84	1.23	1.07
0.9***	1				0.55	0.8	0.88	1.45	1.67
0.79***	0.8***	1			0.5	0.76	0.8	1.54	1.98
0.82***	0.86***	0.84***	1		0.39	0.69	0.86	2.01	4.49
0.77***	0.8***	0.79***	0.85***	1	0.34	0.66	0.77	2.2	5.06

*OASIS*: Overall Anxiety Severity and Impairment Scale, *M*: Mean, *SD*: Standard Deviation, *ITC*: Item-total correlation corrected for scale reliability and item overlap

***p < 0.001

### Convergent and divergent validity, test-retest reliability

The Zero-order bivariate correlation coefficient indicated that there was a strong positive relationship between the OASIS and the GAD-7, the PSS_10, the GSES, and the PHQ_9 (see Table 4). Moreover, there is a medium correlation between the OASIS and the BFI_N and the PHQ_15. Furthermore, a strong negative correlation exists between the OASIS and the RSES and the CORE OM (W, F, P). A medium negative correlation was found between the OASIS and the SWLS and the SCS (Table 4). The first ANCOVA model revealed significantly higher anxiety in females compared to males with a large effect size: $F\left(\right)\left(1,54\right)=8.04$, $p=0.006$, ${\widehat{\eta }}_{G}^{2}=0.130$, 90% CI $\left[0.023,0.275\right]$. However, after the shared variance with neuroticism was partialled out, there was no longer a significant difference in anxiety between males and females: $F\left(\right)\left(1,51\right)=3.97$, $p=0.052$, ${\widehat{\eta }}_{G}^{2}=0.072$, 90% CI $\left[0.000,0.208\right]$. The two-way random effect intraclass correlation coefficient suggested that the OASIS score was sufficiently stable after a 1-week interval: *r* = 0.66, 95% CI [0.13–0.87] *p* = 0.013.

**Table 4:** Spearman correlations between OASIS and other measured constructs

variables	1	2	3	4	5	6	7	8	9	10	11	12	13	14	15	16	17	M	SD
1. OASIS	-																	9.11	4.51
2. RSES	− 0.54***	-																24.21	5.53
3. BFI_N	0.51***	− 0.52***	-															3.73	0.71
4. BFI_A	− 0.19	0.16	− 0.05	-														3.61	0.43
5. BFI_O	− 0.11	0.05	− 0.02	0.27*	-													3.52	0.72
6. PSS_10	0.58***	− 0.65***	0.44***	− 0.41**	− 0.10	-												22.41	6.71
7. SWLS	− 0.38**	0.58***	− 0.39**	0.19	0.12	− 0.48***	-											17.23	7.14
8. GSES	0.56***	− 0.81***	0.41**	− 0.17	− 0.13	0.64***	− 0.46***	-										2.43	0.74
9. CORE_OM_WB	− 0.65***	0.64***	− 0.35**	0.38**	0.16	− 0.76***	0.57***	− 0.60***	-									1.89	0.96
10. CORE_OM_FUNC	− 0.57***	0.58***	− 0.28*	0.55***	0.29*	− 0.66***	0.66***	− 0.53***	0.78***	-								2.15	0.75
11. CORE_OM_prob_symp	− 0.64***	0.55***	− 0.41**	0.47***	0.08	− 0.59***	0.61***	− 0.47***	0.71***	0.77***	-							2.05	0.79
12. SCS	− 0.47***	0.69***	− 0.44**	0.27	0.13	− 0.49***	0.55***	− 0.74***	0.57***	0.57***	0.57***	-						2.62	0.70
13. PHQ_15	0.46**	− 0.49**	0.15	− 0.10	− 0.13	0.44**	− 0.48***	0.35*	− 0.36*	− 0.37*	− 0.44**	− 0.15	-					10.36	5.10
14. GAD_7	0.68***	− 0.63***	0.30*	− 0.34*	− 0.01	0.62***	− 0.46***	0.61***	− 0.65***	− 0.66***	− 0.82***	− 0.49***	0.52***	-				9.30	5.41
15. PHQ_9	0.56***	− 0.74***	0.24	− 0.33*	− 0.15	0.65***	− 0.64***	0.67***	− 0.63***	− 0.71***	− 0.76***	− 0.63***	0.57***	0.77***	-			2.24	0.76
16. Age	0.02	0.28*	− 0.33*	0.07	− 0.14	− 0.10	− 0.03	− 0.20	0.13	0.04	0.05	0.24	0.30*	− 0.03	0.03	-		35.56	14.95
17. Education	− 0.09	0.32*	− 0.08	0.01	0.06	− 0.14	0.18	− 0.16	0.17	0.19	0.02	0.08	− 0.31*	0.00	− 0.17	0.06	-	3.18	0.83
18. Religiosity	− 0.01	− 0.02	0.12	− 0.14	− 0.24	− 0.04	0.04	0.11	− 0.04	− 0.08	0.08	− 0.22	− 0.39**	− 0.16	− 0.13	− 0.34*	− 0.22	2.45	0.69

*OASIS* Overall Anxiety Severity and Impairment Scale, *RSES *Rosenberg Self Esteem Scale, *BFI_N *Big Five Inventory - Neuroticism subscale, *BFI_A *Big Five Inventory - Agreeableness subscale, *BFI_O *Big Five Inventory - Openness subscale, *PSS_10 *Perceived Stress Scale, *SWLS *Satisfaction With Life Scale, *GSES *Guilt and Shame Experience Scale, *CORE_OM_WB *Clinical Outcomes in Routine Evaluation - Well-being subscale,* CORE_OM_FUNC *Clinical Outcomes in Routine Evaluation - Functioning subscale, *CORE_OM_prob_symp *Clinical Outcomes in Routine Evaluation - Problems and Symptoms subscale, *SCS *Self-Compassion Scale, *PHQ_15 * Patient Health Questionnaire Somatic Symptom Severity Scale, *GAD_7 *Generalized Anxiety Disorder Questionnaire, *PHQ_9 *Patient Health Questionnaire

### Cut-off points identification

The evaluation of the cut-off point for the CSC revealed that the optimal score for discrimination between the clinical and non-clinical populations is 5.27. In order to avoid pathologization of clients - we have rounded this value up (6), not down (5). In the second step, the cut-off point for the RCI was estimated. This estimation resulted in a value of 5.33.

In the final step, we aimed to create the cut-offs differentiating people suffering from mild, moderate and severe anxiety based on standard deviations from the median. Analysis of these cut-off points on the Sample 1 indicated that the OASIS score depicting mild anxiety ranges from 5 to 12, moderate anxiety from 13 to 16 and severe anxiety from 17 to 20.

## Discussion

This study aimed to psychometrically examine the Czech version of the OASIS and create population norms. We found a higher OASIS score in females, religious non-church members, widows or widowers, administrative workers, employees in services, pensioners, and students. The CFA supported the unidimensional solution of the OASIS. The measurement equivalence examination suggested that the OASIS measures anxiety invariantly between males and females. The validity of the OASIS was supported via negative correlations with self-compassion, well-being and self-esteem and via positive correlations with depression, symptoms of somatic diseases, perceived stress, and the established anxiety measure. The results also revealed that the internal consistency of the OASIS was excellent and the test-retest reliability was satisfactory.

This study found that the OASIS measures anxiety equivalently between males and females on configural, metric scalar, and strict levels. This finding aligns with the results of the previous study [66] examining measurement invariance between males and females. Thus, the present study provides further support that differences between the two sexes can be most likely attributed to actual differences in anxiety rather than to differences related to other factors such as biased responding [67].

The results regarding the dimensionality of the OASIS indicated that a one-factor structure has an optimal fit. This is in line with the original study results [16]. The present study found the correlated error variance between items 1 and 2 that was also found in previous studies [9, 15, 66, 68, 69]. This might be because the first two items depend on each other. Item 1 asks about frequency, and item 2 asks about the intensity of anxiety symptoms during the past week. So, the absence of symptoms in item 1 leads to the absence of their intensity in item 2.

The convergent validity of the OASIS was supported by a strong positive correlation with the CORE OM P and a well-established anxiety measure. This is in line with previous studies, which also found a strong positive relationship between the OASIS and the established measures of anxiety [11, 17, 66]. In our study, we also found that the OASIS shows significant positive correlations with depression or stress levels that generally overlap with anxiety [15]. In addition, similar to previous studies [70], there was found a medium positive correlation between the OASIS and somatic symptoms, which can be explained by common physical symptoms of anxiety disorders [71].

In the present study, we have found a significant negative correlation between self-compassion and the OASIS. To our knowledge, the present study is the first to examine the relationship between the OASIS and the SCS. Our results are in line with the theoretical assumptions of Neff [36], who proposed that self-compassion should be positively associated with mental health outcomes such as fewer symptoms of depression or anxiety and greater life satisfaction. Similarly, [72] proposed that an accepting attitude toward one’s affect can attenuate distress.

A strong negative correlation between the OASIS and self-esteem was found in our study. Some previous studies [11, 66] also found a negative relationship between self-esteem and the OASIS. Our findings are in line with the theoretical accounts of Greenberg et al., [73], suggesting that higher self-esteem should provide protection against anxiety.

We also found that the threshold of 6 points on the OASIS should be applied to capture the CSC. Therefore, a decrease below 6 points could be regarded as a clinically significant improvement in practice. In contrast, Hermans et al., [17] mentioned a 2-point lower value of clinically significant change in their studies. This distinction could be caused by different measured scores in the Czech clinical and non-clinical samples. In addition, there were diverse ways of rounding, rounded 4.41 to 4, and [17] rounded 3.32 to 4. In this context, we have chosen a more conservative approach that may partially explain the higher value.

Bragdon et al. [18] determined the OASIS cut-off scores for clinician-rated severity of anxiety. In their study the scores of 6, 10, and 12 indicated moderate, marked, and severe disorder severity. On the other hand, we determined the OASIS cut-off scores of 5, 13, and 17 to indicate mild, moderate, and severe anxiety levels in the Czech population. In addition to the explanations mentioned above, the higher cut-off scores in our study could be caused by the pandemic situation in recent years.

### Strengths and limitations

Due to its excellent psychometric properties, comprehensive Czech norms, and ease of completion, the Czech version of the OASIS is suitable for screening in primary and secondary care or continuous mapping of the client’s anxiety severity and impairment. Furthermore, due to the above-mentioned characteristics and the defined cut-offs for the CSC and the RCI, it is also a useful measure for research purposes (e.g., in outcome studies).

There were some limitations to this study. First, the data collection was conducted during the COVID-19 pandemic. The increased prevalence of affective disorders and anxiety disorders specifically during these circumstances, could have influenced the identified cut-off scores in the OASIS. Second, the government policies in health facilities to prevent the spread of COVID-19, including quarantine restrictions, significantly complicated data collection. One consequence is that recruiting samples took place at different times. Given that the prevalence of anxiety in the population may have varied during this turbulent period, we consider the different times of samples recruiting as one of the limitations of the study. Third, the number of participants who filled out retest questionnaires was low. Therefore, the test-retest reliability coefficient may not be sufficiently accurate. Fourth, the measures used for the OASIS validity testing were all self-report scales and questionnaires, which can lead to a social desirability bias. The OASIS validity could be supported by using behavioral or psychophysiological measures. Fifth, the internal consistency of the BFI-A was unsatisfactory. This might be the primary reason why we did not find a significant association between the OASIS and agreeableness.

## Conclusion

The OASIS represents a reliable and valid instrument for assessing anxiety in the adult population. Due to its shortness, excellent psychometric properties, and percentile norms, it is especially useful for short and accurate screening of anxiety and mapping therapeutic change in clinical practice.

## Supplementary Information

**Additional file 1.**

## Data Availability

The datasets generated and/or analysed during the current study are available in the Open Science Framework (https://osf.io) repository, 10.17605/OSF.IO/VPTB5. On this repository, there can also be found the Czech version of the Overall Anxiety Severity and Impairment Scale (OASIS) as well as the study code and preregistration form.
